# Different Acute Kidney Injury Patterns after Renal Ischemia Reperfusion Injury and Extracorporeal Membrane Oxygenation in Mice

**DOI:** 10.3390/ijms231911000

**Published:** 2022-09-20

**Authors:** Robert Greite, Johanna Störmer, Faikah Gueler, Rasul Khalikov, Axel Haverich, Christian Kühn, Nodir Madrahimov, Ruslan Natanov

**Affiliations:** 1 Clinic for Nephrology and Hypertension, Hannover Medical School, 30625 Hannover, Germany; 2 Clinic for Cardiac, Thoracic, Transplantation and Vascular Surgery, Hannover Medical School, 30625 Hannover, Germany; 3 Clinic for Cardiac Surgery, University Hospital Würzburg, 97080 Wurzburg, Germany

**Keywords:** acute kidney injury, extracorporeal membrane oxygenation, heme oxygenase-1, renal inflammation

## Abstract

The use of extracorporeal membrane oxygenation (ECMO) is associated with acute kidney injury (AKI) in thoracic organ transplantation. However, multiple other factors contribute to AKI development after these procedures such as renal ischemia-reperfusion injury (IRI) due to hypo-perfusion of the kidney during surgery. In this study, we aimed to explore the kidney injury patterns in mouse models of ECMO and renal IRI. Kidneys of C57BL/6 mice were examined after moderate (35 min) and severe (45 min) unilateral transient renal pedicle clamping and 2 h of veno-venous ECMO. Renal injury markers, neutrophil infiltration, tubular transport function, pro-inflammatory cytokines, and renal heme oxygenase-1 (HO-1) expression were determined by immunofluorescence and qPCR. Both procedures caused AKI, but with different injury patterns. Severe neutrophil infiltration of the kidney was evident after renal IRI, but not following ECMO. Tubular transport function was severely impaired after renal IRI, but preserved in the ECMO group. Both procedures caused upregulation of pro-inflammatory cytokines in the renal tissue, but with different time kinetics. After ECMO, but not IRI, HO-1 was strongly induced in tubular cells indicating contact with hemolysis-derived proteins. After IRI, HO-1 was expressed on infiltrating myeloid cells in the tubulo-interstitial space. In conclusion, renal IRI and ECMO both caused AKI, but kidney damage after renal IRI was more pronounced including severe neutrophil infiltration and tubular transport impairment. Enhanced HO-1 expression in tubular cells after ECMO encourages limitation of hemolysis as a therapeutic approach to reduce ECMO-associated AKI.

## 1. Introduction

Extracorporeal membrane oxygenation (ECMO) is increasingly used in the context of thoracic organ transplantation [1,2,3]. Veno venous (vv) ECMO is applied in severe respiratory failure refractory to intense mechanical ventilation and provides extracorporeal lung support and veno arterial (va) ECMO is used for cardiac support [4]. Both strategies have been successfully used as a bridge to lung transplantation [5]. Acute kidney injury (AKI) complicates ECMO treatment in up to 63% of patients [6] and similar AKI rates have been reported in the context of vv and va ECMO [7]. Severe AKI requiring dialysis is associated with a 3.7-fold increase in risk of mortality in ECMO-patients [6]. The underlying mechanisms of AKI-development in the context of ECMO therapy are complex and multifactorial including co-morbidity-related factors, inflammation and administration of nephrotoxic drugs [8]. A key factor affecting AKI risk is renal ischemia reperfusion injury (IRI) due to hemodynamic instability and episodes of kidney hypo-perfusion which is common in patients at need of ECMO therapy [3]. Although ECMO can be a life-saving therapy, the ECMO system per se can also put patients at risk of AKI due to hemolysis from red blood cell breakdown [9]. Dissection of these multifactorial mechanisms of AKI in ECMO patients in the setting of clinical studies is challenging. Examination of kidney tissue by biopsy in patients with AKI while on ECMO-support is not feasible due to the high bleeding risk associated with anticoagulation in ECMO therapy [10]. Therefore, kidney tissue analysis in ECMO treatment is limited to animal models which are scarce and commonly performed in large animals [11]. However, recently, we demonstrated technical feasibility of the vv ECMO system in mice which offers the opportunity to investigate the mechanism of kidney injury through the ECMO system itself in the absence of an underlying disease or emergency condition [12].

Since renal IRI and ECMO-associated kidney damage often occur together in the clinical context of organ transplantation [6], the aim of this study was to characterize the kidney injury pattern in a mouse model of ECMO and renal IRI.

## 2. Results

### 2.1. Renal NGAL Expression

**Figure 1:**
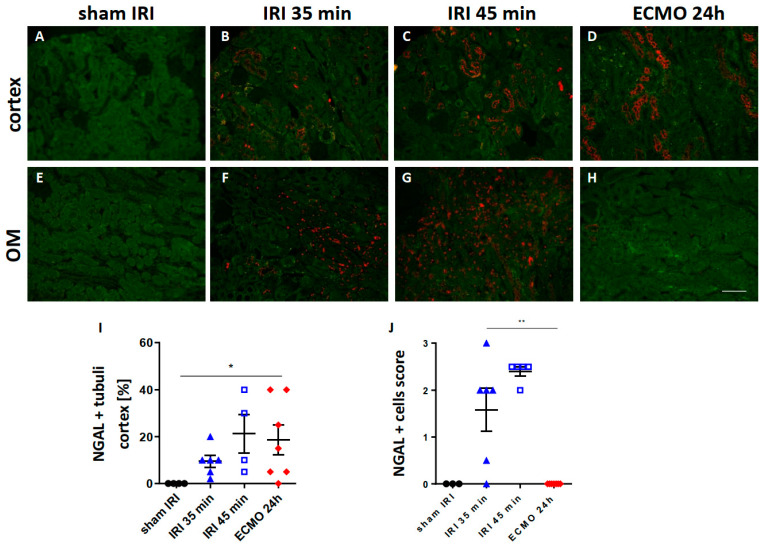
Renal injury marker NGAL in the kidney after moderate and severe renal IRI compared with ECMO. The renal cortex (upper row) and outer medulla (lower row) of NGAL-stained kidney sections are shown 24 h after moderate (**B**,**F**) and severe (**C**,**G**) renal IRI and 24 h (**D**,**H**) after ECMO. The NGAL staining signal is shown in red, tubular autofluorescence in green. Sham-IRI mice and sham-ECMO mice had no significant tubular NGAL expression and sham-IRI control is shown for convenience (**A**,**E**). Scoring of tubular NGAL expression is shown for the renal cortex in (**I**) and the semi-quantitative scoring of NGAL-expressing cells in the outer medulla (OM) is shown in (**J**). ECMO and severe renal IRI both caused enhanced tubular NGAL expression in the renal cortex (**C**,**D**,**I**). In the outer medulla which is most vulnerable to hypoxia increased NGAL-expressing cells were found after moderate (**F**) and severe (**G**) renal IRI which were absent in ECMO-treated mice (**H**). N = 4–7 mice each group are shown. * *p* < 0.05, ** *p* < 0.01. Scale bar 100 µm.

To overcome early mortality, a unilateral renal IRI model was applied in this study. Therefore, the most common kidney function marker serum creatinine is not meaningful in this setting, because the contralateral healthy kidney compensates for the loss of renal function caused by the unilateral injury and serum creatinine levels remain normal. To overcome this limitation, immunofluorescence staining for NGAL in the injured kidney was performed which was shown to correlate with urinary NGAL and detected AKI in a previous study [13]. Both procedures, moderate and severe renal IRI and ECMO, caused increased positivity for NGAL in tubular cells of the renal cortex (Figure 1B–D,I). Moreover, NGAL represents one of the neutrophil secondary granule proteins and is highly expressed in neutrophils [13,14,15]. In the outer medulla, severe interstitial infiltration of NGAL-expressing cells was present after moderate and severe renal IRI, but not ECMO, suggesting that these cells are neutrophils (Figure 1F,G,J).

### 2.2. Kidney Neutrophil Infiltration

**Figure 2:**
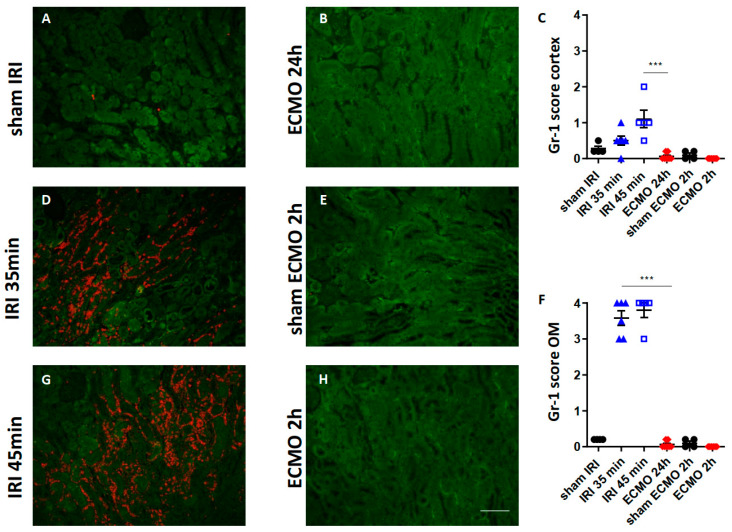
Neutrophil infiltration of the kidney after moderate and severe renal IRI and after ECMO. Gr-1-stained kidney sections of mice 24 h after moderate (**D**) and severe (**G**) renal IRI and 2 h (**H**) and 24 h (**B**) after ECMO are shown. The Gr-1 staining signal is shown in red, tubular autofluorescence in green. Sham-IRI mice and sham-ECMO mice served as controls (**A**,**E**). Semi-quantitative scoring of Gr-1 expression is shown for the renal cortex in (**C**) and for the outer medulla (OM) in (**F**). Moderate and severe renal IRI caused marked infiltration of the ischemic kidney with neutrophils (orange signal, (**D**,**G**)) whereas no major renal neutrophil infiltration 2 h (**H**) or 24 h (**B**) after ECMO was observed. N = 4–7 mice each group are shown. *** *p* < 0.001. Scale bar 100 µm.

So far, both procedures caused renal upregulation of pro-inflammatory cytokines. Therefore, infiltration of the kidney with neutrophils was determined by immunofluorescence for Gr-1. Moderate and severe renal IRI both caused marked infiltration of the renal cortex and outer medulla with neutrophils (Figure 2C,D,F,G). In contrast, neither 2 h nor 24 h after ECMO treatment was neutrophil infiltration of the kidney detectable (Figure 2B,C,F,H). Sham surgery caused no significant neutrophil infiltration (Figure 2A,C,E,F).

### 2.3. Tubular Transport Function

**Figure 3:**
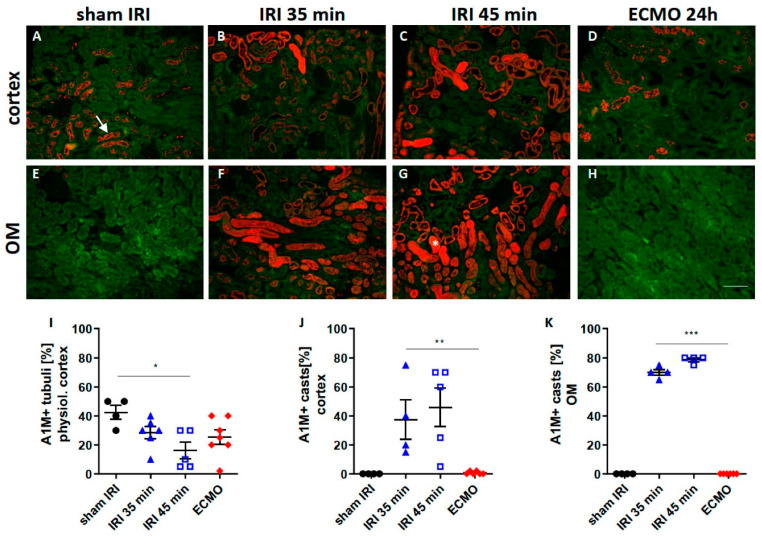
Staining for the tubular transport marker A1M in the kidney after moderate and severe renal IRI compared with ECMO. The renal cortex (upper row) and outer medulla (lower row) of A1M-stained kidney sections are shown 24 h after moderate (**B**,**F**) and severe (**C**,**G**) renal IRI and 24 h (**D**,**H**)) after ECMO. The A1M staining signal is shown in red, tubular autofluorescence in green. Sham-IRI mice and sham-ECMO mice had similar A1M staining and sham-IRI control is shown for convenience (**A**,**E**). Physiologically, a tubular granular staining pattern of A1M can be observed ((**A**), white arrow). In conditions of tubular transport impairment, the granular tubular staining pattern is reduced and A1M accumulates in the tubular lumen forming casts ((**G**), white asterisk). Moderate and severe IRI were associated with a reduction in the physiologic A1M signal (**I**) and an increase in A1M cast formation (**B**,**C**,**F**,**G**,**J**,**K**) indicating impairment tubular transport function. ECMO did not cause A1M cast formation (**D**,**H**,**J**,**K**) indicating preserved tubular transport function. N = 4–7 mice each group are shown. * *p* < 0.05, ** *p* < 0.01, *** *p* < 0.001. Scale bar 100 µm.

A1M is physiologically expressed in a granular pattern in renal tubular epithelial cells. Disruption of tubular transport capacity leads to accumulation of A1M in casts in the tubular lumen which indicates tubular transport impairment [16]. Severe renal IRI led to a significant decrease in the physiological granular A1M pattern (Figure 3C,I). Pathological intra-luminal formation of A1M positive casts was markedly increased after renal IRI in the cortex (Figure 3B,C,J) and outer medulla (Figure 3F,G,K). After ECMO, only physiological A1M signal and no pathological formation of A1M positive casts was evident (Figure 3D,H–K) indicating preserved tubular transport function after ECMO.

### 2.4. Time Kinetics of Kidney Tissue Pro-Inflammatory Cytokines

**Figure 4:**
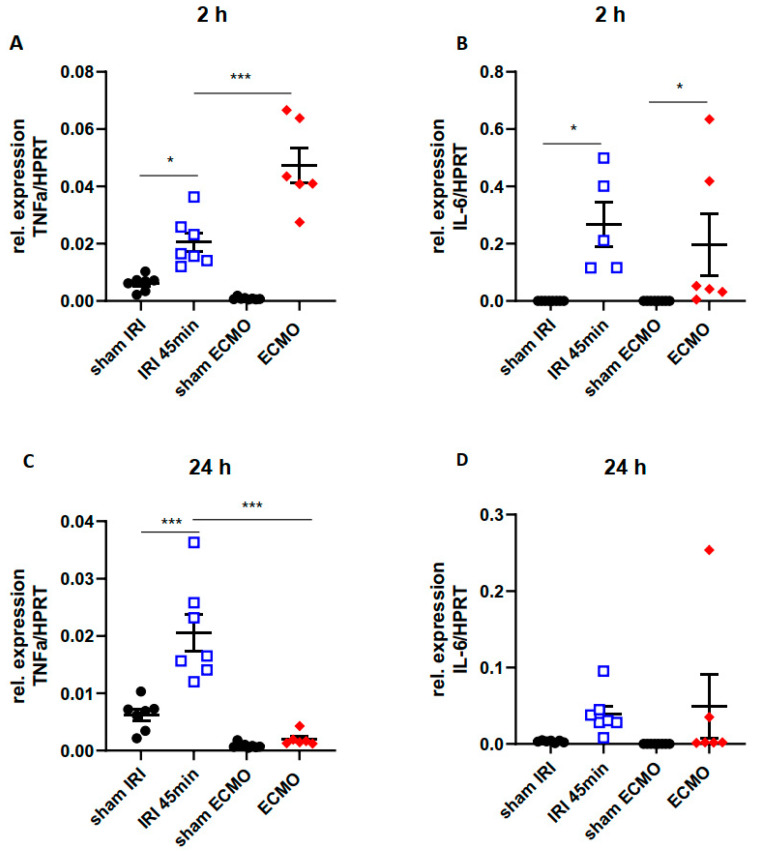
Pro-inflammatory cytokines in the kidney tissue 2 h and 24 h after renal IRI and ECMO. The pro-inflammatory cytokines TNFα (**A**,**C**) and IL-6 (**B**,**D**) were determined by qPCR in the renal tissue 2 h (**A**,**B**) and 24 h (**C**,**D**) after renal IRI for 45 min (blue bordered squares) and ECMO for 2 h (red diamond suits). Sham-IRI mice (opening of the abdominal cavity without manipulation of the renal vessels) and sham-ECMO mice (cannulation without connection to the extracorporeal circuit) had no significant upregulation of renal TNFα or IL-6 levels (black dots). Renal upregulation of TNFα was more pronounced 2 h after ECMO (**A**) compared with renal IRI and normalized after 24 h (**C**). In renal IRI however, TNFα expression in the kidney increased after 2 h compared with sham (**A**) and was significantly higher compared with ECMO mice after 24 h (**C**). Early enhanced IL-6 expression was observed in renal IRI and ECMO (**B**) which was decreased 24 h after both procedures (**D**). N = 5–8 mice each group are shown. * *p* < 0.05, *** *p* < 0.001.

Kidney tissue levels of the pro-inflammatory cytokines TNFα and IL-6 were determined by qPCR 2 h and 24 h after renal IRI for 45 min and ECMO for 2 h. Sham surgeries served as control. After ECMO, TNFα elevation was markedly higher 2 h after surgery compared with renal IRI (Figure 4A) and normalized after 24 h (Figure 4C). However, after 24 h increase in TNFα kidney tissue levels was significantly higher following renal IRI compared with ECMO (Figure 4C). IL-6 increased 2 h after renal IRI and ECMO (Figure 4B) and returned to normal after 24 h (Figure 4D). In summary, both procedures induced upregulation of pro-inflammatory cytokines in the kidney, but with different time kinetics.

### 2.5. Renal HO-1 Expression

**Figure 5:**
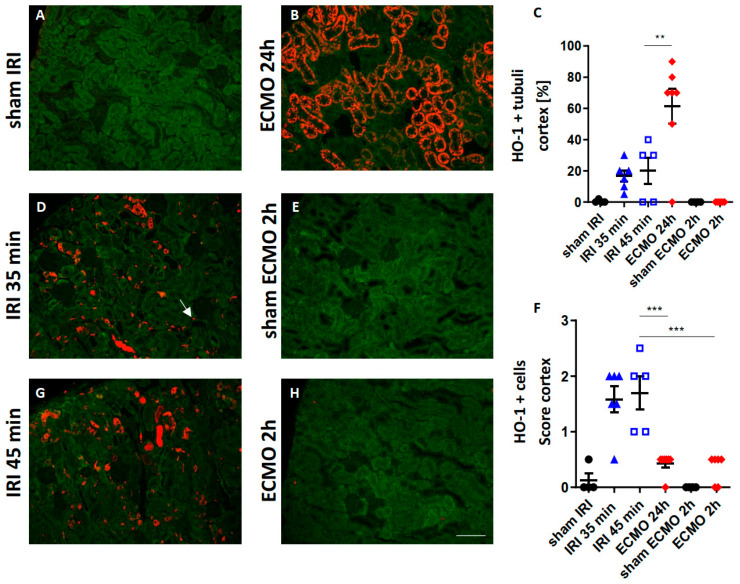
Renal HO-1 expression after moderate and severe renal IRI compared with ECMO. HO-1-stained kidney sections of mice 24 h after moderate (**D**) and severe (**G**) renal IRI and 2 h (**H**) and 24 h (**B**) after ECMO are shown. The HO-1 staining signal is shown in red, tubular autofluorescence in green. Sham-IRI mice and sham-ECMO mice served as controls (**A**,**E**). Two hours after ECMO no renal HO-1 expression was evident (**E**). However, 24 h after ECMO strong tubular upregulation of HO-1 was observed (**B**,**C**). Moderate and severe renal IRI only caused minor tubular HO-1 expression (**D**,**G**,**C**). However, after renal IRI the extent of HO-1 expressing cells (white arrow) was significantly higher in the renal cortex compared with ECMO (**F**). ** *p* < 0.01, *** *p* < 0.001. Scale bar 100 µm.

HO-1 can be expressed in renal tubular epithelial cells and leukocytes in response to oxidative stress [17] and hemolysis [18,19]. Macrophages are the pre-dominantly HO-1 expressing cells [19]. HO-1 expression in the kidney was determined by immunofluorescence after moderate and severe renal IRI and different time-points after ECMO. ECMO caused significantly enhanced expression of HO-1 after 24 h in tubular epithelial cells of the renal cortex compared with renal IRI (Figure 5B–D,G). However, following moderate and severe renal IRI the number of HO-1 expressing cells in the tubule-interstitial space was markedly higher (Figure 5B,D,F,G). In summary, HO-1 expression in the kidney following renal IRI and ECMO revealed a different pattern: After ECMO, HO-1 was expressed in tubular epithelial cells, whereas after IRI it was expressed in infiltrating tubulo-interstitial leukocytes.

## 3. Discussion

Although AKI can complicate ECMO therapy in up to 63% of patients [6], the molecular mechanisms and morphological kidney damage pattern are less known. Renal IRI as a consequence of transient intraoperative hypotension is associated with increased AKI risk after lung transplantation [20]. Since both conditions, renal IRI and ECMO, can synergistically contribute to AKI development, this study thought to explore the kidney injury pattern in mouse models of unilateral renal IRI and vv ECMO.

To overcome early mortality associated with bilateral renal IRI in mice [21], models of unilateral renal IRI with 35 min and 45 min transient renal ischemia were chosen in this study. These renal ischemia times have been shown to induce moderate and severe AKI in a previous study [22]. Serum creatinine cannot be used to monitor renal function after unilateral renal IRI, because the contralateral unclipped kidney compensates for loss of renal function. Therefore, we determined the expression of the injury marker NGAL in renal tubular epithelial cells, which correlates with urinary NGAL and indicate AKI, in a previous study [13]. Severe renal IRI and ECMO both caused enhanced tubular NGAL expression in the renal cortex indicating that both procedures caused AKI. In the outer medulla of the kidney, an increased number of NGAL-expressing cells was found in the tubulo-interstitial space after moderate and severe renal IRI, but not after ECMO. NGAL is highly expressed by neutrophils as part of the of the neutrophil secondary granule proteins [13,14,15]. Indeed, we found enhanced neutrophil infiltration after renal IRI, which was absent after ECMO. This neutrophil infiltration was most pronounced in the outer medulla of the kidney after renal IRI. A possible explanation can be that the oxygen tension is lowest in the renal outer medulla which makes this region most vulnerable to ischemic injury [23]. In contrast, a previous study showed that vv ECMO in mice for 4 h caused a slight increase in neutrophil infiltration in the glomeruli and tubule-interstitial space [24]. However, in our study no major renal neutrophil infiltration was observed after ECMO treatment for 2 h which might be explained by the shorter duration of ECMO treatment.

To study the kidney injury patterns after renal IRI and ECMO, the vv and not va ECMO system was applied in this study because va ECMO was shown to promote tissue hypoxia by impairment of microvascular perfusion which caused ischemic kidney damage in a previous study [25]. At a first glance, this is contradictory, since va ECMO is applied to improve overall organ perfusion in conditions of cardiogenic shock [4]. However, the blood flow generated by the va ECMO system is continuous [26] and it was demonstrated previously that a pulsatile and not continuous blood flow is important to preserve renal perfusion [26,27,28]. Since vv ECMO does not alter arterial flow, a pulsatile flow of the native heart is generally maintained during vv ECMO [26]. However, a recent study in miniature pigs found decreased renal artery flow and urine output as well as signs of ischemic tubular injury and mitochondrial dysfunction after vv ECMO [29]. Importantly, renal blood flow was measured after 24 h of vv ECMO and not during the vv ECMO procedure due to technical feasibility in the study by Szabó-Biczók et al. [29]. However, these authors maintained the mean arterial pressure during vv ECMO above 60 mmHg which is known to preserve renal perfusion [29,30]. Szabó-Biczók and colleagues concluded that the development of AKI precedes the hemodynamic changes in their mini pig model of vv ECMO [29]. As in their study, measurement of renal artery flow during vv ECMO was also not technically feasible in our miniaturized mouse ECMO system. Thus, changes in renal hemodynamics during vv ECMO will be elucidated in future studies.

Ischemic kidney injury is associated with altered tubular transport function indicated by reduced physiological expression of A1M by renal tubules [16]. Impairment of tubular function has also been shown in a mouse model of cardiopulmonary bypass [31] and urinary A1M has been postulated as a biomarker for AKI after cardiopulmonary bypass surgery in patients [31,32]. However, so far, A1M and tubular transport function have not been investigated in ECMO treatment. In this study, we found that vv ECMO did not alter tubular transport function in contrast to renal IRI.

ECMO and renal IRI can both cause release of pro-inflammatory cytokines into the circulation [33,34]. In a porcine ECMO model, plasma levels of TNFα increased 2 h after ECMO [35]. Moreover, a strong increase in systemic IL-6 levels in ECMO treatment was observed in previous studies [33,36,37] and persistent IL-6 elevation during ECMO was associated with increased mortality [38]. However, studies on pro-inflammatory cytokine expression by the kidney in response to ECMO are scarce. A study by Yimin et al. investigated renal pro-inflammatory cytokines 24 h after ECMO and found a significant increase in TNFα and IL-6 in the renal tissue [39]. In our ECMO-model, we found an early increase in kidney tissue TNFα and IL-6 2 h after ECMO which was transient and normal cytokine levels were observed 24 h after ECMO. In contrast, the time kinetics in renal IRI were different with a pronounced TNFα-increase 24 h after surgery.

Moreover, HO-1 expression in renal tubular cells was markedly pronounced following ECMO, but not after renal IRI. Upregulation of HO-1 in renal tubular cells has been described in settings of hemolysis [40]. Cell-free hemoglobin is oxidized to methemoglobin during hemolysis and the pro-oxidant heme is released. Free heme that is not bound to proteins has major pro-inflammatory and cytotoxic effects [41,42,43]. HO-1 is responsible for intracellular degradation of toxic free heme [44]. The association of ECMO and hemolysis in previous studies [9] and the enhanced tubular HO-1 expression after ECMO we found in this study suggests a potential mechanistic link of hemolysis and AKI after ECMO. A limitation of the current study is that the enhanced tubular HO-1 expression is only an indirect indicator of hemolysis. Future studies measuring markers of hemolysis in the murine model of ECMO will elucidate if the enhanced HO-1 expression after ECMO is a consequence of hemolysis, e.g., free heme release. In contrast to the HO-1 expression in renal tubular cells following ECMO, HO-1 was expressed on cells located in the tubulo-interstitial space after renal IRI. Previous studies have shown that HO-1 is expressed on a different myeloid cells including neutrophils, dendritic cells, and macrophages after renal IRI [19]. Moreover, HO-1 expressing myeloid cells promoted resolution of inflammation and recovery in ischemic AKI [17].

Taken together, this study shows that renal IRI and ECMO both cause AKI in this model. However, the injury pattern was markedly different between renal IRI and ECMO. Renal IRI was associated with severe tubular injury, tubular transport impairment, and infiltration of the kidney with neutrophils. The vv ECMO system caused mild signs of tubular injury and did not alter tubular transport function or cause neutrophil infiltration in this model. Enhanced expression of HO-1 by renal tubular cells following ECMO indicates that hemolysis might cause AKI in this ECMO model in otherwise healthy mice. In conclusion, approaches limiting hemolysis in ECMO treatment might reduce ECMO-associated kidney injury.

## 4. Materials and Methods

### 4.1. Animals

Male, 8–10 weeks old, C57BL/6 mice purchased from Charles River (Sulzfeld, Germany) were used for the experiments. All mice were kept under a 14/10 h day/night cycle and had free access to drinking water and food. All experiments were approved by the local animal protection committee of the Lower Saxony State department for animal welfare and food protection (14/1657, 16/2250). Mice were monitored daily for their physical condition after surgery. Reasons for study termination were visible behavioral changes such as scrubby appearance, reduced motility, reduced food uptake, reduced activity, or body weight reduction of >20%. Moderate unilateral IRI for 35 min was performed in n = 6 mice. Severe unilateral renal IRI for 45 min was performed in n = 5 mice. Sham surgery in the IRI experiments was performed in n = 4 mice. ECMO treatment was performed in n = 6 mice with sacrifice 2 h after ECMO disconnection and in n = 7 mice with sacrifice at 24 h after ECMO disconnection. Sham surgery in the ECMO experiments was performed in n = 4 mice.

### 4.2. Renal Ischemia Reperfusion Injury (IRI)

**Figure 6:**
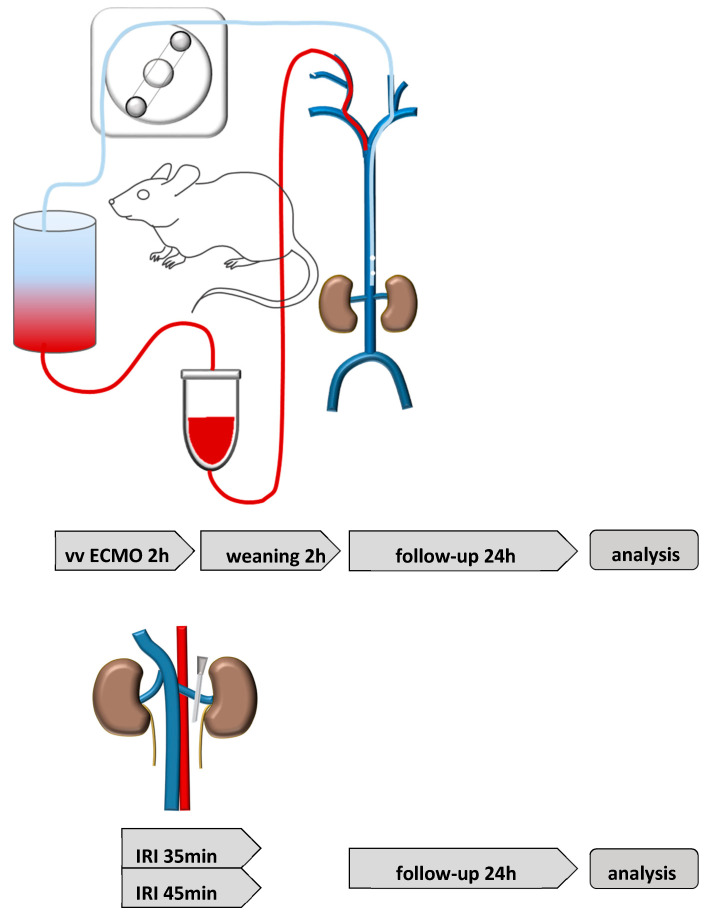
Experimental set-up. The vv ECMO (**upper row**) was performed by cannulation of the superior vena cava via the right jugular vein (red line) and the inferior vena cava via the left jugular veins (blue line). Mice were kept on ECMO support for 2 h. Weaning from ECMO was performed for additional 2 h. Kidney tissue analysis was performed either after the weaning period or 24 h after weaning from ECMO. Unilateral renal IRI (**lower row**) was performed for 35 min or 45 min and analysis was performed 24 h after renal IRI.

Isoflurane was administered for anesthesia (3% induction, 1–2% maintenance) and butorphanol (1 mg/kg) for analgesia. IRI was induced by unilateral renal pedicle clamping with a microaneurysm clip (Aesculap, Tuttlingen, Germany) for 35 min (moderate IRI) or for 45 min (severe IRI). Reperfusion was controlled visually. Sham surgery was performed by opening of the abdominal cavity, but without manipulation of the renal vessels. The experimental set-up is shown in Figure 6.

### 4.3. vv Extracorporeal Membrane Oxygenation (ECMO)

The ECMO circuit was designed as previously described [45]. The re-designed micro-oxygenator (200 μL) and air-trapping chamber (150 μL) contained a smaller priming volume, thus reducing the total priming volume to below 500 μL. Prior to the surgical procedures, the ECMO circuit was primed with 500 µL of a 1:1 solution of Tetraspan and Sterofundin (B Braun Medical, Melsungen, Hesse, Germany) that had been heparinized with 30 IU/mL of perfusion solution and buffered with 2.5% *v*/*v* of an 8.4% solution of sodium bicarbonate. After priming, this solution was left circulating until ready for cannulation. Two lateral neck skin incisions were made and both jugular veins were exposed. The cranial segments of both veins were ligated cranial to the bifurcation and slip knots were placed at the proximal segments of the vessel. Using a 2 Fr. intravenous cannula, connected to the inflow tubing, cannulation of the right jugular vein was performed and advanced 5 mm towards the superior vena cava. Subsequently, the left jugular vein was exposed and animals were fully heparinized (2.5 IU/g body weight) via direct intravenous injection. For cannulation of the left jugular vein, two fenestrations were made in the distal part of a 2 Fr. polyurethane tube. The cannula was then inserted through the left jugular vein into the inferior vena cava shortly proximal of the iliac bifurcation. Previous measurements allowed placing the draining part of the cannula directly between the renal veins and the iliacal bifurcation. For real-time invasive blood pressure monitoring, as well as arterial blood sampling, the left femoral artery was cannulated with a 1-Fr polyurethane cannula and connected to a pressure transducer. Sham animals underwent similar anesthesia and analgesia. After anesthesia induction, the preparation of both jugular veins was performed as previously described. After cannulation of both veins, the animal was kept in narcosis for the time of the experiment, but no extracorporeal circulation was initiated. Mice were kept for either 2 or 24 h post operation. The experimental set-up is shown in Figure 6.

### 4.4. Physiological Monitoring during ECMO

For physiological monitoring during the procedure, electrocardiography (ECG), rectal temperature (in degrees Celsius) and invasive blood pressure (in mmHg) via catheterization of the left femoral artery were used. These probes were then connected to a multichannel data acquisition system (Hugo Sachs Elektronik GmbH, March, Germany) and analyzed using the ISOHEART Software (Version 1.5.7, Harvard Apparatus Inc., Holliston, MA, USA). Routine blood gas analysis (BGA; iSTAT, Abbot Laboratories Inc., Chicago, IL, USA) was conducted by the sampling of venous and arterial blood to control the proper oxygenation and metabolic state of mice. During the experiment, the rate of anticoagulation was controlled by measuring the activated clotting time (ACT). The ACT was kept between 160 and 180 s.

### 4.5. Organ Preservation

Mice were sacrificed in deep general anesthesia (5% isoflurane) 2 h and 24 h after ECMO disconnection or sham surgery and 24 h after moderate and severe renal IRI or sham surgery and organ retrieval was performed. After midline laparotomy, whole-body perfusion with ice-cold 0.9% PBS via the cannulated left ventricle resulted in a circulatory arrest. Organs were dissected and fixed in RNA later or 4% paraformaldehyde.

### 4.6. Pro-Inflammatory Cytokine Expression in the Kidney Tissue

Total mRNA was isolated from cross-sectioned kidney slices using RNeasy Mini Kit (Qiagen, Hilden, Germany) and cDNA was subsequently synthetized with Prime Script Reverse Transcriptase reagent (Takara, Kusatsu, Japan) from DNase-treated total RNA. A LightCycler 96 (Roche, Penzberg, Germany) was used to conduct qPCR. The following primers were used: tumor necrosis factor alpha (TNFα, Qiagen, #QT00104006) and interleukin-6 (IL-6, Qiagen, #QT00098875). Hypoxanthine phosphoribosyl transferase (HPRT) (Qiagen, #QT00166768) served as housekeeper for normalization.

### 4.7. Renal Morphology and Immunofluorescence

After paraffin embedding, 2 μm sections were cut and immunofluorescence was performed using the following antibodies: Gr-1 antibody for neutrophils (Ly-6G/Ly-6C+, Serotec, Oxford, UK), neutrophil gelatinase-associated lipocalin (NGAL, R&D systems, Minneapolis, MI, USA), alpha-1-microglobulin (A1M, Kind gift from Dr. Magnus Gram, Lund, Sweden), and heme oxygenase-1 (HO-1, Enzo life sciences, New York, NY, USA). Tubular NGAL, A1M, and HO-1 expression were quantified as percentage of the affected tubuli in 10 different areas. The physiological A1M signal was determined as the granular pattern within tubular epithelial cells and pathological A1M signal as A1M expressing intra-luminal tubular cast formation. Neutrophil infiltration, NGAL-expressing cells, and HO-1 expressing cells in the tubulo-interstitial space of the renal outer medulla (OM) were analyzed semi-quantitatively using the following score: 0: <5 cells/view field (VF); 1: 5–10 cells/VF; 2: 11–20 cells/VF; 3: 21–50 cells/VF; and 4: >50 cells/VF. Analysis was conducted on a Leica imaging microscope at 200-fold magnification in 10 different view fields per sample. Investigators were blinded to the group assignment. Images were captured with the same magnification.

### 4.8. Statistical Analysis

Statistical analysis was performed with GraphPad Prism 5.0 (GraphPad Software, San Diego, CA USA, Version 5.0). Multiple comparisons were analyzed through a one-way ANOVA and group means were compared using the Tukey’s post hoc test. Data are reported as mean value ± standard error of the mean (SEM). *p*-values < 0.05 were accepted as significant.

## Data Availability

Original data are available on request from the corresponding author.
